# Response to letter regarding clinical significance of the systemic immune inflammation index in relation to pathology treatment and outcomes in acute appendicitis a retrospective study

**DOI:** 10.1080/07853890.2026.2627684

**Published:** 2026-03-10

**Authors:** Beige Zong

**Affiliations:** Department of General Surgery, Chongqing Emergency Medical Center, Chongqing, China

Thank you for the opportunity to respond to the thoughtful comments raised by Tao et al. regarding our recently published article [1]. We sincerely appreciate their interest in our work and their constructive suggestions, which will undoubtedly help advance future research in this field.

Below, we provide a point-by-point response to their observations:

1. Regarding methodological clarity and exclusion of confounding factors

Our hospital serves as the primary emergency medical center for acute abdominal conditions in the city. Patients who received prior pharmacological interventions (e.g. antibiotics, corticosteroids) at other institutions typically had undergone relevant laboratory tests before transfer, and such hematological data are often unavailable in our electronic medical records. Therefore, cases with missing laboratory values were systematically excluded from our analysis. In addition, the category ‘Other diseases’ was used to exclude chronic conditions such as hypertension, diabetes, coronary heart disease (CHD), and chronic obstructive pulmonary disease (COPD)—which could incur additional inpatient costs—as well as pregnancy, which alters standard clinical management. This category included comorbidities such as chronic non-atrophic gastritis, fatty liver, simple renal cysts, and urinary tract infections, which were not considered to substantially affect hospitalization expenses related to appendicitis management. Besides, upon reexamination of our database, we confirm that no patients with systemic hematological disorders were included in this cohort.

1. Validation of clinical thresholds and ROC analysis

Due to the retrospective nature of our study, C-reactive protein (CRP) and procalcitonin (PCT) were not routinely measured for all patients, resulting in a significant amount of missing data that precluded meaningful ROC curve comparisons. We acknowledged this as a key limitation in the Discussion section.

The SII thresholds mentioned in our study (i.e. 1500, 2000, and 2500) were proposed as hypothetical values based on retrospective clinical observation. Whether these thresholds are applicable in clinical practice requires validation through prospective, multicenter studies. Our primary aim was to lay a foundation for such future investigations. Therefore, we did not perform ROC analysis to determine optimal cutoff values in this study, and hope for your understanding.

1. Comparison with established clinical scoring systems

We agree that direct quantitative comparison between SII and existing clinical scoring systems—such as the Alvarado Score, AIR Score, and the Adult Appendicitis Score—would enhance the clinical relevance of our findings. We thank the readers for this valuable suggestion and will incorporate such comparisons in our planned prospective studies to better demonstrate the incremental utility of SII.

1. Potential integration into primary care and health systems

We are encouraged by the readers’ recognition of SII’s potential, particularly within China’s ‘15minute Medical Circle’ framework. We fully support their recommendation to integrate SII into primary care decision-support tools and to leverage health information platforms for real-time data sharing across different levels of care. These insights will guide our future research directions.

Once again, we express our gratitude to Tao et al. for their insightful comments. Their feedback not only acknowledges the strengths of our study but also provides meaningful guidance for subsequent research. We are committed to refining our methodology and expanding our investigations in line with these constructive suggestions.

## Data Availability

Data sharing is not applicable to this article as no data were created or analysed in this research.
